# Characterization of multi-channel intraneural stimulation in transradial amputees

**DOI:** 10.1038/s41598-019-55591-z

**Published:** 2019-12-17

**Authors:** I. Strauss, G. Valle, F. Artoni, E. D’Anna, G. Granata, R. Di Iorio, D. Guiraud, T. Stieglitz, P. M. Rossini, S. Raspopovic, F. M. Petrini, S. Micera

**Affiliations:** 1 Center for Neuroscience, Neurotechnology, and Bioelectronic Medicine and The BioRobotics Institute, Scuola Superiore Sant’Anna, Pisa, Italy; 2 Bertarelli Foundation Chair in Translational Neuroengineering, Centre for Neuroprosthetics and Institute of Bioengineering, School of Engineering, École Polytechnique Fédérale de Lausanne (EPFL), Lausanne, Switzerland; 3 Fondazione Policlinico Agostino Gemelli-IRCCS, Roma, Italy; 4 Institute of Neurology, Catholic University of The Sacred Heart, Policlinic A. Gemelli Foundation, Roma, Italy; 5 University of Montpellier, INRIA, CAMIN team, 860 Rue St Priest, 34090 Montpellier, France; 6 Laboratory for Biomedical Microtechnology, Department of Microsystems Engineering–IMTEK, Bernstein Center, BrainLinks-BrainTools Cluster of Excellence, University of Freiburg, Freiburg, D-79110 Germany; 7 Laboratory for Neuroengineering, Department of Health Sciences and Technology, Institute for Robotics and Intelligent Systems, ETH Zürich (ETH), Zürich, 8092 Switzerland; 8 Department of Excellence in Robotics & AI, Scuola Superiore Sant’Anna, Pisa, Italy

**Keywords:** Translational research, Peripheral neuropathies

## Abstract

Although peripheral nerve stimulation using intraneural electrodes has been shown to be an effective and reliable solution to restore sensory feedback after hand loss, there have been no reports on the characterization of multi-channel stimulation. A deeper understanding of how the simultaneous stimulation of multiple electrode channels affects the evoked sensations should help in improving the definition of encoding strategies for bidirectional prostheses. We characterized the sensations evoked by simultaneous stimulation of median and ulnar nerves (multi-channel configuration) in four transradial amputees who had been implanted with four TIMEs (Transverse Intrafascicular Multichannel Electrodes). The results were compared with the characterization of single-channel stimulation. The sensations were characterized in terms of location, extent, type, and intensity. Combining two or more single-channel configurations caused a linear combination of the sensation locations and types perceived with such single-channel stimulations. Interestingly, this was also true when two active sites from the same nerve were stimulated. When stimulating in multi-channel configuration, the charge needed from each electrode channel to evoke a sensation was significantly lower than the one needed in single-channel configuration (sensory facilitation). This result was also supported by electroencephalography (EEG) recordings during nerve stimulation. Somatosensory potentials evoked by multi-channel stimulation confirmed that sensations in the amputated hand were perceived by the subjects and that a perceptual sensory facilitation occurred. Our results should help the future development of more efficient bidirectional prostheses by providing guidelines for the development of more complex stimulation approaches to effectively restore multiple sensations at the same time.

## Introduction

Major effort has been devoted to restoring sensory feedback in upper limb amputees^1–15^. Using different sensory encoding and stimulation strategies, information from sensors embedded into hand prostheses has been translated into sensations perceived by the users^1–3,5,7,9,10,13–16^. Through direct neural stimulation using peripheral neural interfaces, various types of sensations have been elicited, such as pressure, touch, vibration, paraesthesia, tingling, or electricity^4,7,8,17^.

Recent studies have shown that these elicited sensations can be used for the real-time control of hand prostheses^2,10,18^. This approach allows sensory feedback to be used in simulated daily living activities^18^ by exploiting multiple sensations through stimulating the median and/ or ulnar nerves^15,18^. This leads to an improvement in motor control^4,17,19^, as well as the ability to recognize the shape and compliance of objects^7,15^ or to simultaneously integrate position and tactile information^20,21^.

In these studies, the sensations evoked through electrical stimulation were characterized by stimulating single active sites (ASs). Further two ASs (one from the median and one from the ulnar nerve) were combined in a multichannel setup. However, to the best of our knowledge no detailed characterization of the stimulation parameters and the sensations evoked for combined-channel configurations has been carried out so far. A deeper understanding of this mechanism could potentially increase the effectiveness of sensory feedback in sensorized prosthetic systems.

**Figure 1:**
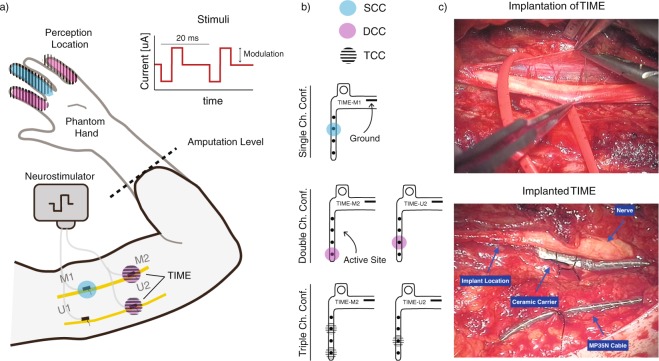
Stimulation set-up. (**a**) Implanted TIMEs in median (M1-2) and ulnar (U1-2) nerves, are connected to the STIMEP or RIPPLE neurostimulator which sends rectangular, bi-phasic and cathodic first stimuli with changing amplitude but constant pulse-width and frequency. Stimulation of M1, M2 and U2 led to somatotopic sensation feedback located on the amputee’s phantom limb. Each electrode used its own GND. (**b**) Schemes of multi-channel configurations SCC, DCC and TCC. (**c**) Insertion of TIME and implanted and fixed TIMEs in ulnar nerve.

In our study, four transradial amputees were implanted with four transverse intrafascicular multi-channel electrodes (TIMEs^22^) . Two were implanted in the median nerve and two in the ulnar nerve (Fig. 1). Sensory feedback was restored to the subjects by stimulating single ASs, as well as combinations of different ASs. Information regarding the location, perceptual threshold and type of these sensations was collected. Sensation properties were analyzed to characterize and compare single-channel (SCC), two-channel (DCC), and three-channel (TCC) stimulation configurations.

## Results

### Sensation characterization

First, we characterized the subjects’ rating of the stimulation of single active sites. 2-second trains of biphasic and charge-balanced current pulses were injected through the implanted TIME active sites. In order to find the perceptual thresholds for each active site (Materials and Methods), the pulse amplitude was modulated between 10 µA and 980 µA, whereas the pulse-width was fixed to between 20 µs and 120 µs. The frequency was fixed to 50 Hz. The interval between each pulse train was 2 seconds. The subjects were asked to report the location, extent, type, and strength of the sensation. The strength of the sensation was rated with a scale from 0 to 10, where 0 was “no sensation”, 1 was the sensation threshold, 10 was pain.

Then, to assess the outcome of DCC and TCC, the ASs that were the most repeatable during SCC (Materials and Methods) in terms of reported sensation properties, were selected for combination. DCC stimulation took place with one AS stimulating in the median, and one AS in the ulnar nerve, or both ASs in one nerve. For TCC, two ASs of the median nerve and one AS for the ulnar nerve were used (Table 1 and Fig. Supp. 1). The pulses of current were simultaneously delivered to the active sites in multi-channel configurations. Here we show the results of the ASs that were used in SCC, DCC, TCC.

**Table 1:** SCC, DCC and TCC combinations.

	SCC	DCC	TCC
Subject 1	• M2 AS5, • M2 AS6, • M2 AS14, • U1 AS11, • U1 AS12	• M2 AS5 + U1 AS12, • M2 AS5 + U1 AS11, • M2 AS6 + U1 AS11, • M2 AS14 + U1 AS11	• M2 AS14 + M2 AS 5 + U1 AS12 • M2 AS14 + M2 AS 5 + U1 AS11
Subject 2	• M2 AS12, • M2 AS4, • M1 AS2, • M1 AS3, • U2 AS1, • U2 AS8	• M2 AS4 + U2 AS8, • M2 AS12 + U2 AS1, • M1 AS2 + M2 AS4, • M1 AS3 + M2 AS4	
Subject 3	• M1 AS1, • M1 AS5, • M2 AS4, • U1 AS4, • U2 AS2, • U2 AS4	• M1 AS1 + U1 AS4, • M2 AS4 + U2 AS4, • M1 AS5 + U1 AS4, • M2 AS4 + U2 AS2	
Subject 4	• M1 AS7, • M2 AS7, • M2 AS14, • U1 AS7, • U1 AS12, • U1 AS13	• M2 AS14 + U1 AS13, • M2 AS14 + U1 AS7, • M2 AS7 + U1 AS12, • M2 AS12 + U1 AS12, • M1 AS7 + U1 AS12	• M2 AS7 + U1 AS1 + U1 AS12 • M2 A14 + U1 AS1 + U1 AS12

**Figure 2:**
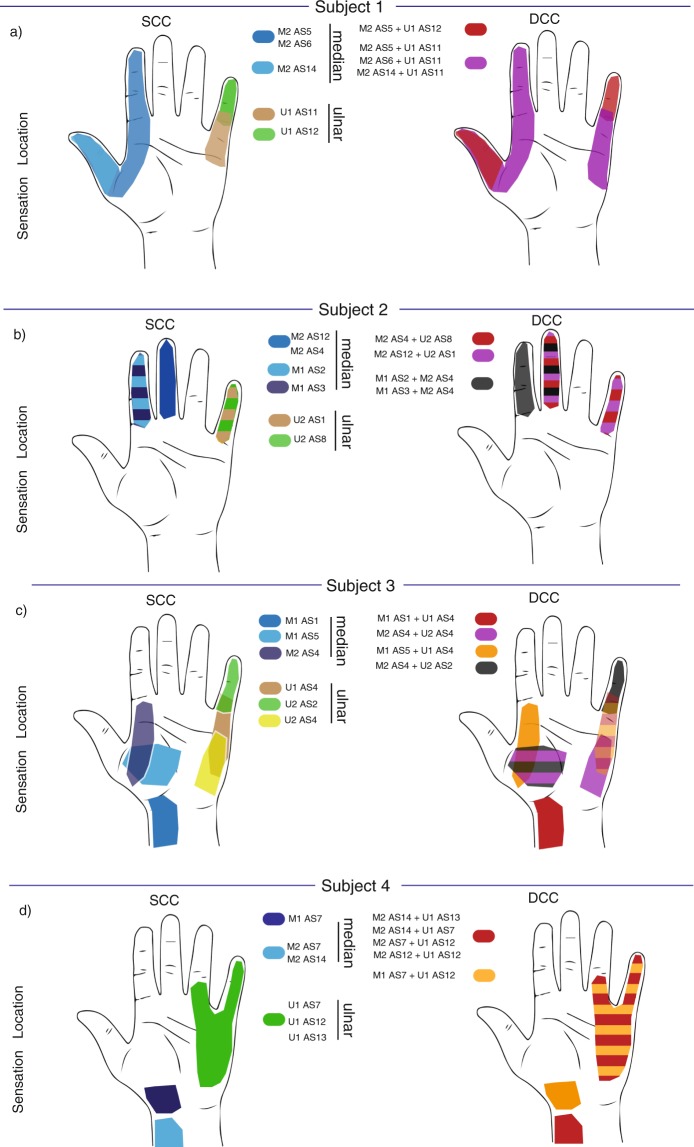
SCC and DCC sensation location. Left side: evoked sensation location during single channel, intraneural stimulation (SCC) with TIMEs. Right side: double channel configuration (DCC). (**a**) Subject 1, (**b**) Subject 2, (**c**) Subject 3, (**d**) Subject 4.

The elicited sensation locations for SCC and DCC for all four subjects are shown in Fig. 2. Thumb, index and little phantom finger were perceived by subject 1 (Fig. 2a). During DCC (Fig. 2b), two channels used together caused a combination of the sensation locations perceived during SCC (e.g., thumb plus little finger, simultaneously). This linear combination was observed for all the double-channel configurations.

The results for subject 2 (Fig. 2b) show that the SCC stimulation elicited sensations on the index, third, and little phantom fingers. As in subject 1, when combining the stimulation channels (DCC), the linear combination of the SCC sensations was perceived. For subjects 3 and 4, the results were similar to those for subjects 1 and 2 (Fig. 2c,d). Subjects 1 and 2 also reported that there was occasionally a non-linear summation of the single-channel stimulation sensations. They reported sensations whose location was different from the one elicited through single-channel stimulations (e.g. two channels eliciting, one channel stimulated a sensation on the phantom thumb, and the other a sensation on the phantom little finger, generated a sensation on the phantom palm).

**Figure 3:**
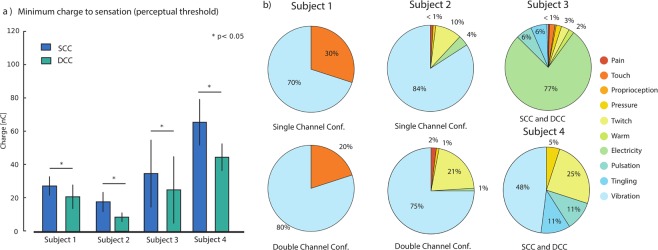
SCC and DCC perceptual threshold. Perceptual threshold for all subjects in respect to injected charge for SCC and DCC. Y-axis shows the average single-contact charge in SCC and DCC configurations, where x indicates subject 1–4. All the data showed significant differences (p < 0.05) between SCC and DCC (N = 30, 162, 30, 33, for Subject 1, 2, 3 and 4, respectively).

The minimum charge to elicit a sensation for every subject with SCC and DCC stimulations is shown in Fig. 3 and Fig. Supp. 2. For all the subjects, a higher charge was needed per single active site to reach the perceptual thresholds when stimulating in SCC compared to DCC. The average charges used for subjects 1–4 was 37.72nC in SCC, and 16.58nC in DCC. Hence in DCC, 11nC less were applied, which corresponds to a 37.28% reduction. Statistical significance (p < 0.05) was shown for all four subjects.

The perceived sensation types related to all four subjects for SCC and DCC are shown in Fig. 3b. For subject 1, touch was reported when stimulating with a SCC in 30% of the cases, while vibration was reported in 70% of the cases. Stimulating with a DCC, there was 20% touch and 80% vibration. Subject 2 reported similar sensations in both cases. In SCC: vibration 85%, warm feeling 10%, electricity 4%, pressure <1% and cold <1%. In DCC vibration 75%, warm feeling 21%, cold 2%, electricity <1%, pressure <1%. For Subject 1 and Subject 2 the occurrence of the sensation types in single- and multi-channel configurations were not statistically different (p > 0.01). Subject 3 reported mainly electricity and the proportion remained the same in SCC and in DCC (p = 1). The same happened with subject 4, the most perceived sensation being vibration (p = 1).

**Figure 4:**
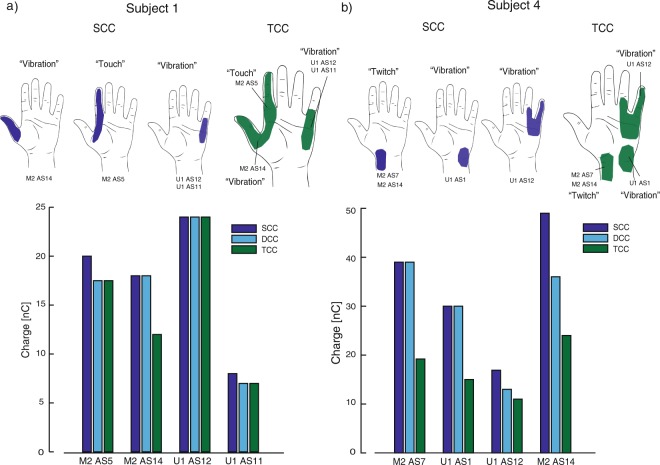
TCC characterization. Sensation location for subject 1 (**a**) and 4 (**b**) for SCC (blue) and TCC (green). Bottom: Perceptual thresholds for subject 1 (**a**) and 4 (**b**) during SCC, DCC and TCC (N = 42, 45, for Subject 1 and 4, respectively). Y-axis: single active site charge level (threshold) needed to elicit a sensation in each stimulation condition. X-axis: ASs which have been used in SCC, DCC and TCCs.

When stimulating in TCC (Subjects 1 and 4), the sensation location and type, as in DCC, were the result of the linear combination of the sensations evoked in SCC (Fig. 4). The sensation thresholds obtained with TCC (Fig. 4) were even lower than the ones obtained with DCC, which were lower than in SCC (reported above). For subject 1 the charge needed to reach perceptual thresholds, decreased by 9% from DCC to TCC, and by 5% from SCC to DCC. For subject 4 there was a decrease of 41% from DCC to TCC, and 22% from SCC to DCC.

### EEG characterization

EEG data was acquired from Subject 1 in order to further investigate SCC, DCC, and TCC stimulations. Single channel stimulation was performed for both the median and ulnar nerves, double channel stimulation through one active site of the median nerve and one of the ulnar nerve, and three-channel stimulation with two ASs of the median nerve and one AS of the ulnar nerve. Sub-perceptual threshold (SS) was tested as the control condition for the median nerve stimulation.

**Figure 5:**
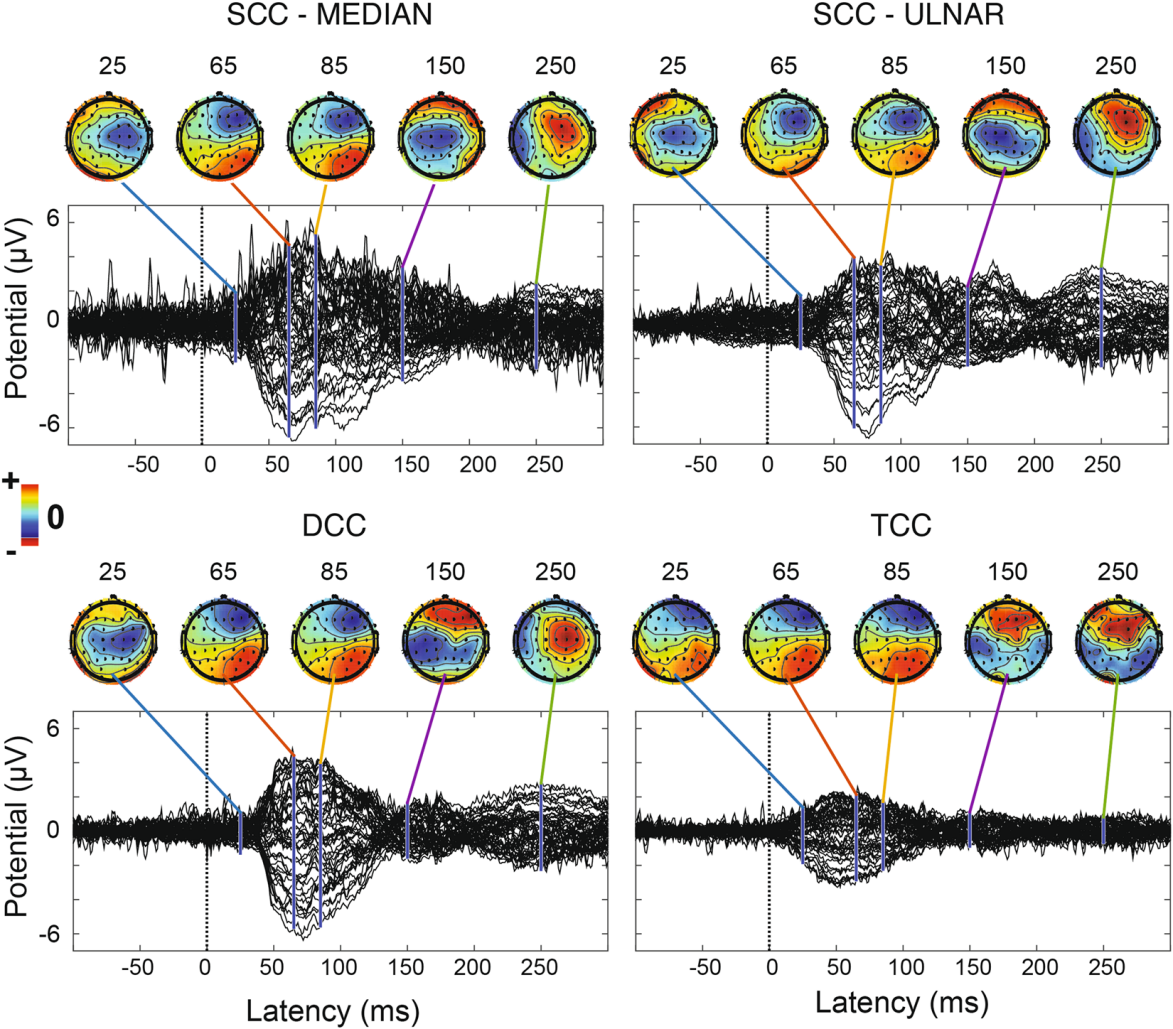
SCC, DCC and TCC cortical recordings. Subject 1 grand-average butterfly SEPs and topographic scalp maps at different latencies for SCC-median, SCC-ulnar, DCC and TCC conditions (respectively N = 412, 349, 427 and 337). Latencies are referred to the onset of the stimulation.

**Figure 6:**
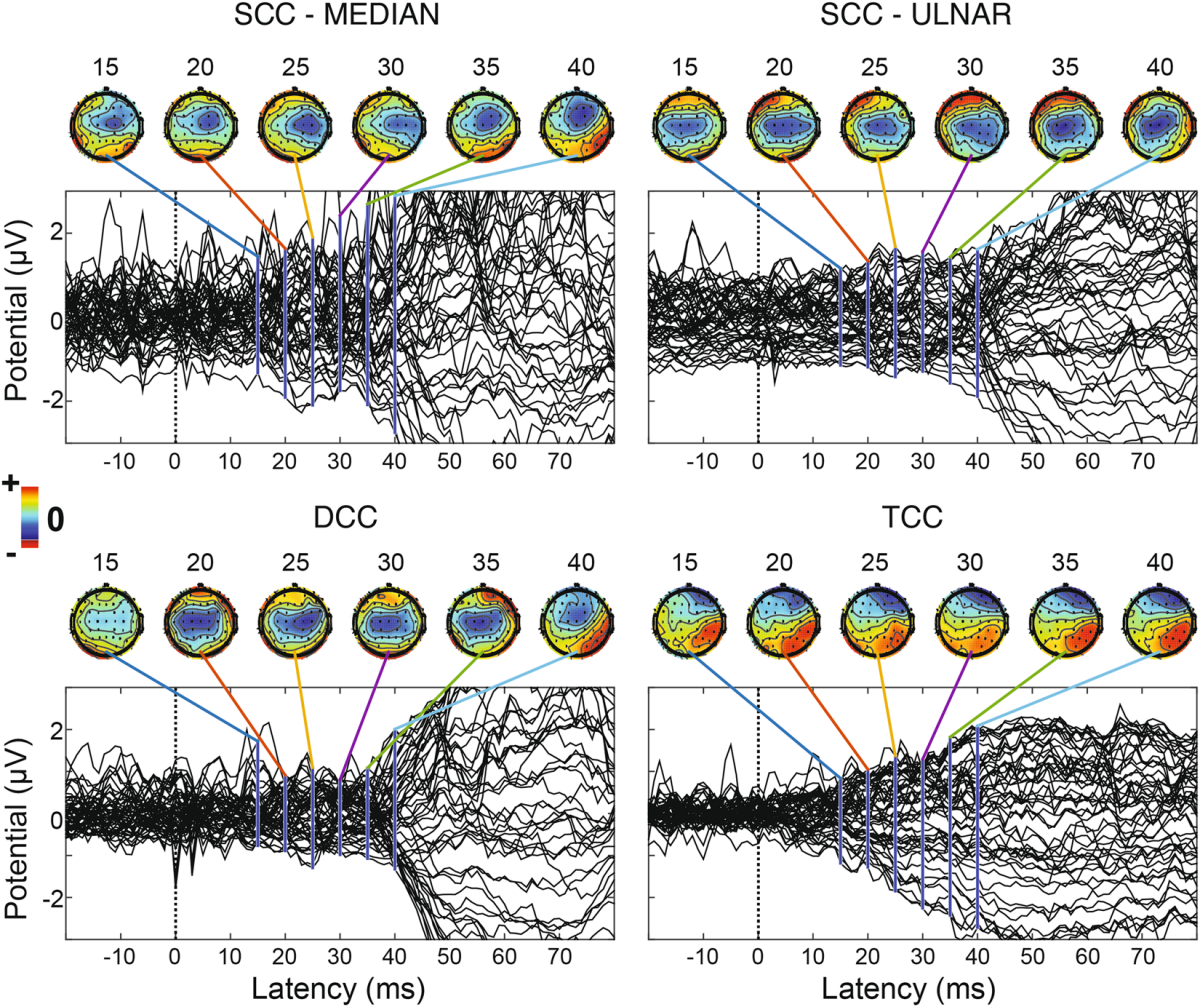
Early latencies zoom on grand-average butterfly SEPs. Early latencies zoom on grand-average butterfly SEPs for Subject 1. As in Fig. 5, topographic scalp maps are shown at different latencies respectively for SCC-median, SCC-ulnar, DCC and TCC conditions (respectively N = 412, 349, 427 and 337). Latencies are referred to the onset of the stimulation.

Figure 5 shows the grand-average somatosensory evoked potentials (SEPs) for subject 1 at late latencies. Scalp topographies for each condition show that 60–80 ms after the onset of the stimulation there was a clear contralateral activation. Furthermore, all conditions exhibited an activation as early as 15–20 ms (Fig. 6) after the onset of the stimulation (N20). In TCC, this happens more contralaterally and with lower amplitude (Figs. 5 and 6) with respect to SCC and DCC. In fact, the N20 TCC scalp map is similar to the SCC, DCC and TCC scalp maps at later latencies. As expected, sub-perceptual threshold stimulation (Fig. Supp. 3) elicited no significant response. Figure Supp. 4 shows a clear SEP distributed mainly contralaterally across the scalp. Statistical analyses performed over three contralateral EEG derivations (F4 – frontal, C4 – central and P4 – posterior, see Figure Supp. 4) confirm a significantly lower amplitude (p < 0.05) of TCC SEPs with respect to DCC, SCC-median and SCC-ulnar.

## Discussion

In order to characterize multi-channel stimulation configurations for restoring sensory feedback in prosthetic users, four subjects were implanted with TIMEs in their median and ulnar nerves. We characterized these configurations both through subjective patient reports, and objective EEG measures.

### Sensation characterization

In all the subjects, the sensation type remained the same in SCC, DCC and TCC, and the location of the sensation obtained in multi-channel configurations turned out to be the combination of the sensation location elicited in single-channel configurations.

In fact, locations and types of sensations depend on the number and types of sensory fibers elicited by the neural stimulation^23^. As expected, by stimulating with two electrodes implanted in two different nerves, the fiber types addressed and numbers did not differ with respect to SCC. Interestingly, even when stimulating two electrodes that were placed in the same nerve (Subject 2), results were similar to when DCC involved two nerves.

It has been reported that when sensations from multiple points of the phantom are evoked at a very similar intensity, the subject has the illusion of perceiving only one sensation, which is the combination of the multiple points^24^. However, this effect was anecdotally reported only by Subjects 1 and 2. We hypothesize that these reports were sporadic because during the characterization process, the sensations elicited were not always of exactly the same intensity^25^. This further shows that the TIMEs in combination with the neurostimulators create clear sensations with low crosstalk between channels^26^. Indeed, we excluded that the perception of a single central sensation relates to the summing of voltage fields from multiple channels resulting in activation of different neural populations than in single channel configurations, since the stimulation channels used were at the distance higher than 1.6 mm^27^ (apart for one TCC combination).

The thresholds of the injected charge reveal that DCCs caused a significant reduction in the threshold charge compared to the same channels used with SCCs. This result would seem to indicate a perceptual facilitation due to the simultaneous stimulation of two residual nerves. It has been proposed that the somatosensory cortex is not just a simple relay, but can actively contribute to the integration of somatosensory inputs^28^. For the use of two or more channels together in a closed-loop neuroprosthesis, the right perceptual threshold must be set in order to correctly exploit the multiple sensory feedback during daily life activities.

### EEG characterization

The EEG analysis showed that the tactile sensations generated by SCC, DCC and TCC stimulation elicited early SEP components. They are characterized by positive and negative deflections respectively on the contralateral parietal (e.g., P2, P4, CP2 and CP4) and frontal (e.g., F2, F4, FC2 and FC4) EEG derivations symmetrically distributed across the CP1- C4 line (Fig. Supp. 4). Scalp topographies of early SEPs (20–80 ms) are compatible with a post-central gyrus (Broadmann areas 2 and 3) dipole, and are consistent with a physiological activation of the primary and secondary somatosensory cortices^11,29,30^. This finding is in line with Forss *et al*.^31^, Maguiere *et al*.^32^ and Allison *et al*.^33^ in terms of median nerve stimulation, and reflects the event cascade that follows the processing of a somatosensory input.

Although contamination by non-somatotopic in-loco sensations cannot be ruled out, the elicited SEPs are compatible with the literature and are significantly different between the various types of stimulation, which suggests a representation of touch in the brain compatible with the referred sensation reported by the subject. By ”touch” we mean sensations due to afferent fibers whose endings were connected to mechanoreceptors before the amputation.

With DCC, the SEP amplitudes do not match the arithmetic sum of median and ulnar nerve stimulation. Similarly, Okajima *et al*.^34^ observed differences between the arithmetical sum of independent right, left, median nerve SEPs. They also observed that the simultaneous stimulation of two nerves evoked SEPs even at early latencies, the greatest difference being at 100 ms after the onset of the stimulation. It is reasonable to suppose that, with two- and three-channel stimulation, there is an interaction between the afferences coming from the arm that influences the evoked potentials: sensory information coming from the hand is integrated at several levels of the sensory pathway, including the brainstem and the thalamus^35^, however cortical processing, perception, phase summation and habituation phenomena might have also come into play.

The TCC SEPs exhibit a smaller amplitude, which further suggests the presence of an early integration process similar to that of DCC. In addition, the amplitude distribution over the scalp channels of N20 for TCC (Fig. 6) is very similar to that of SCC and DCC at later latencies (Fig. 5), which may further corroborate the hypothesis of an early integration of stimuli when performing multi-channel stimulation. In particular, the median nerve stimulation was perceived to be stronger than the ulnar one. This was reported both in SCC and DCC. This predominance is likely to have resulted in DCC scalp topographies slightly more similar to SCC-median than SCC-ulnar. The lower amplitude of DCC and TCC SEPs with respect to the sum of SCC SEPs might also occur due to a “surround inhibition” effect, as observed by Gindrat *et al*.^36^. The sensation perceived by the subject was quite different than median-SCC, ulnar-SCC and DCC. Considering this phenomenon, it could be reasonable that the late components of SEP were different in shape and amplitude respect the other kind of stimulations. Moreover, since the TCC was performed for last, we cannot exclude that an effect of habituation might have come into play and reduced the SEP amplitude. However, the smaller amount of charge (up to a 41% reduction) needed for TCC to elicit a perceivable sensation might also have been a contributing factor for the observed reduced SEP amplitude.

### Prospective applications

Taking into consideration the decrease in thresholds when using SCC, with respect to DCC and TCC, a characterization of all three stimulation configurations should be performed to adapt the injected charges to different types of hand grasps (e.g., pinch, three-digit, precision, and power grasp). Moreover, complex algorithms using multi-channel configurations that can stimulate more than three ASs at a time could be developed to achieve more efficient stimulation strategies^17^.

Our study revealed that single AS thresholds change with the configuration type (SCC, DCC, or TCC) and so the characterization of the sensation has to be repeated for each configuration. To avoid this, an interleaved stimulation delivered through the different channels could be applied^37^.

Finally, to verify the effectiveness of sensory feedback systems that provide stimulation through more than three electrode channels, further experiments are required.

## Materials and Methods

### Subject recruitment

Four transradial amputees were recruited for the clinical trials. Subject 1 was a 36-year-old male, who had undergone a left transradial amputation around 10 years before the experiments. Subject 2 was a 37-year-old male with a two-year old traumatic (car accident) transradial amputation (distal third of his dominant left forearm) two years before the clinical trials. Subject 3 was a 53-year-old female amputee with a transradial amputation at the proximal third of her left arm, caused by an industrial accident. Subject 4, a 48-year-old female with traumatic transradial amputation of the distal third of her left forearm (dominant) occurred 23 years before the trials.

Ethical approval was granted by the Institutional Ethics Committees of the Policlinic Agostino Gemelli at the Catholic University of Rome, Italy. The protocols were approved by the Italian Ministry of Health. Informed consent was signed by all patients. Throughout the entire duration of our study, all experiments were conducted in accordance with relevant guidelines and regulations. Informed consent was given for the publication of identifying information/images.

### Surgical procedure

The implantation methods are described elsewhere^7^. In brief, a general anaesthesia was performed, during which an approximately 15 cm-long incision on the left arm was performed. After exposing the median and ulnar nerves, a microscope was used to implant one proximal and one distal TIME in each nerve (Fig. 1). Electrodes 1 (M1) and 2 (M2) were implanted into the median nerve, whereas electrodes 3 (U3) and 4 (U4) were located in the ulnar nerve. Figure 1b shows the insertion process of the TIME as well as an implanted TIME into the ulnar nerve. Each TIME had 14 AS and two grounds (GNDs), which were used to deliver an electrical current into the nervous tissue. To have access to the electrodes, four co-helical lead cables that were attached to each TIME exited the patient’s arm through four small incisions. To prevent infection, the transcutaneous path was disinfected daily and cleaned. After the experimental trials, the microelectrodes were removed.

### Sensation characterization

To perform a characterization of the sensations evoked through electrical stimulation, a neurostimulator (STG4008, Multichannel Systems, Germany for Subject 1, STIMEP, University of Montpellier and Axonic, France for Subject 2^38^; Ripple Grapevine LLC, USA for Subjects 3, and 4) was connected to the TIME electrodes.

The patients sat in a relaxed position with their stump on a cushion, while electrical pulses with varying amplitude and pulse-width were injected through different ASs of the four TIME electrodes. An emergency button was provided so that the patient could stop the stimulation at any time. Charge-balanced, biphasic, cathodic-first, rectangular stimulation pulses were applied. Single channels (SCC) or multi-channel (DCC and TCC) stimulation configurations were applied (Supp. Fig. 1). All stimuli were applied against the GND of the stimulating electrode. The current was injected into the nerve using one AS against the GND on one electrode (SCC), two ASs against two GNDs on two electrodes (DCC), or three AS against two GNDs on two electrodes (TCC) see Fig. 1 and Supp. Fig. 1. The pulse amplitude was changed between 10 μA and 980 μA with increments of 10 μA, while the pulse-width was fixed between 20 μs and 120 μs, depending on the AS. The stimulation train frequency was set at 50 Hz as in our previous work^7^.

The interval between each pulse train was two seconds. After every stimulus, the subjects were asked to report the sensation location, extent, type and intensity. A custom-made software application was used to control the stimulator and to record stimulation parameters and subjects’ reports. When the amplitude was modulated, the perceived sensation intensity varied accordingly^39^. Since the perceptual thresholds varied between active sites, above all due to the position inside the nerve^17^, they were unknown a priori. Thus, we first set the pulse width at minimal value (e.g. 20 µs) and increased the stimulation amplitude in order to find the perceptual thresholds (from 10 µA to 980 uA). Then, if the perceptual thresholds were not found, we repeated the procedure with a higher pulse width. The perceptual threshold was the average of the charges at which the minimum sensation was reported by the subject. Location and extent were indicated on a picture of a hand, shown by the graphical user interface of our software. The type of the sensation was described by the subjects along with the other parameters and recorded through the software. At the end of the sensation characterization procedure, we found all the charges necessary to reach the perceptual thresholds for each active site. To assess the outcome of DCC and TCC, the ASs that were the most repeatable ones in the SCC characterization procedure were selected to be combined and used. Most repeatable ASs are the ones that do not or only slightly change during the characterization process in terms of reported sensation location, type and perceptual threshold. Trains of stimulation with increasing amplitude were delivered simultaneously from two (DCC) or three (TCC) active sites. The charge range identified during SCC characterization was used for each channel. Then, the threshold of the channels in DCC and TCC was the value of charge at which all the channels themselves elicited a sensation of minimum intensity at the same time. To determine such values, the minimum amplitude of stimulation of all the channels was slightly adjusted. As in SCC the single values of stimulation were delivered at least 3 times. For example: M2 AS5 (SCC) caused a sensation on the index finger. U1 AS12 caused a sensation on the little finger. When combining the two DCC, using the same thresholds as in SCC, the little finger was not perceived. By increasing the minimum delivered amplitude of current of U1 AS12 both sensations could be felt simultaneously. The sensations elicited by DCC and TCC were characterized with the same strategy as in SCC, i.e. by assessing the sensation location, type and perceptual thresholds. The tests took place for a one-month period for Subject 1, and five months for Subjects 2–4 who were implanted in different time periods. The four subjects were implanted over a time course of five years (2013–17). The following stimulation configurations were included: for subject 1 five SCCs and four DCCs were tested. With Subjects 2 and 3 six SCCs and four DCCs were tested, and with Subject 4 six SCCs and five DCCs were tested. TCC was tested with Subjects 1 and 4 only (2 active site combinations each). Table 1 reports the list of combinations tested

### EEG and SEP

Neural correlates of mono and multi-channel stimulation were investigated by acquiring 64-channel electroencephalographic (EEG) data. Subject 1 underwent one rest session (5 minutes) and several stimulation sessions, SCC-median, SCC-ulnar, DCC, and TCC lasting around 10 minutes each. During the recordings, the subject sat on a comfortable armchair and was instructed to maintain his facial muscles relaxed and avoid sudden head movements as much as possible.

In the SCC-median condition, a 20nC charge was injected through AS M2 AS5 with reference to the GND of the same electrode. For the SCC-ulnar, 24nC were injected in U1 AS12. For the DCC, U1 AS12 24nC were applied, whereas M2 AS5 a charge of 17.5nC was injected (different GND for U1 and M2). For the TCC we used M2 AS5 a 17.5nC, U1 AS12 24nC and M2 AS14 with an injected charge of 12nC. M2 AS5 and M2 AS14 were referred to the same GND (median electrode). U1 used the GND of the ulnar electrode.

The inter-stimulus interval was set to 850 ms. Signals were recorded using a 64 channel EEG amplifier (Brain Products BrainAmp) with a 1000 Hz sampling rate. A montage in accordance with the 5% 10/20 system^40^ was used, and electrode impedance was below 10kΩ in at least 95% of recordings throughout the whole experiment.

The data was analyzed using custom Matlab (R2016b, The Mathworks, US) scripts based on routines from the EEGLAB toolbox^41^. Continuous data was processed using a reliable independent component analysis (RELICA) approach^42^ to remove artifacts and other non-neural noise sources, without any preliminary data^43^ dimensionality reduction. To maximize the stability and dipolarity^44^ of independent components (ICs), raw data was high-pass filtered using a zero-phase 1.2 Hz, 24th order Chebyshev type II filter, low-pass filtered using a zero-phase 45 Hz, 70th order Chebyshev type II filter and resampled at 250 Hz. Channels were removed that had a kurtosis outside five standard deviations with respect to other channels or with prominent prolonged artifacts as confirmed by visual inspection. Epochs with high-amplitude artifacts or high- frequency muscle noise were also identified by visual inspection and removed. The remaining data was submitted to RELICA with AMICA core^42,45^, and 100 point-by-point Infomax independent component analysis (ICA)^46^ bootstrap repetitions.

ICA unmixing weights were then re-applied to the raw data, this time high-passed filtered using a 0.5 Hz 94th order Chebyshev type II filter and a custom (complex poles only) 50 Hz comb notch filter^47^. Dipolar and stable ICs related to stereotyped artifacts such as movement and blinks were then removed from the data. Epochs from −100 ms to 300 ms with respect to the onset of each stimulation pulse were extracted and centered with respect to the pre-stimulus baseline. Similarly to continuous data, noisy epochs were rejected by careful visual inspection. The remaining trials for each condition were averaged to yield somatosensory event related potentials (SEPs). Scalp topographies were drawn by color-coding SEP values for each channel location at a particular latency based on amplitude (red – positive values, blue – negative values, green – null values).

### Statistical analysis

All sensation characterization data was extracted and processed in Matlab (R2016b, The Mathworks, US). The normality of the data was first checked (one-sample Kolmogorov-Smirnov test). Since none of the data was normally distributed, a Kruskal-Wallis test was adopted (instead of ANOVA). When necessary, a Tukey-Kramer correction for multi-group comparison was applied. α was set to 0.05. In order to compare the occurrences of sensation types, a Fisher’s test was run. α was set to 0.05. Regarding EEG analysis, significance of SEP differences across the stimulation types (SCC-median, SCC-ulnar, DCC and TCC) for selected channels was assessed using Montecarlo statistics with multiple comparisons cluster correction^48^ adapted from the FieldTrip toolbox^49^.

## Supplementary information

Supplementary material

## Data Availability

The datasets generated during and/or analysed during the current study are available from the corresponding authors on reasonable request.
