# Supplementary material for: Characterization of multi-channel intraneural stimulation in transradial amputees
Source: Sci Rep. 2019 Dec 17;9:19258. doi: 10.1038/s41598-019-55591-z (PMC6917705; doi:10.1038/s41598-019-55591-z)
Supplement: Supplementary file 1 — Supplementary material [file 41598_2019_55591_MOESM1_ESM.docx]

**Characterization of multi-channel intraneural stimulation in transradial amputees**

I. Strauss^1,2,+^, G. Valle^1,2,+^, F. Artoni^2^, E. D’Anna^2^, G. Granata^3^, R. Di Iorio^4^, D. Guiraud^5^, T. Stieglitz^6^, P. M. Rossini^3,4^, S. Raspopovic^7^, F. M. Petrini^2,7,&,*^, S. Micera^1,2,&,*^

^1^ Center for Neuroscience, Neurotechnology, and Bioelectronic Medicine and The BioRobotics Institute, Scuola Superiore Sant'Anna, Pisa, Italy.

^2^ Bertarelli Foundation Chair in Translational Neuroengineering, Centre for Neuroprosthetics and Institute of Bioengineering, School of Engineering, École Polytechnique Fédérale de Lausanne (EPFL), Lausanne, Switzerland.

^3^ Fondazione Policlinico Agostino Gemelli-IRCCS, Roma, Italy.

^4^ Institute of Neurology, Catholic University of The Sacred Heart, Policlinic A. Gemelli Foundation, Roma, Italy.

^5^ University of Montpellier, INRIA, CAMIN team, 860 Rue St Priest, 34090 Montpellier, France.

^6^ Laboratory for Biomedical Microtechnology, Department of Microsystems Engineering–IMTEK, Bernstein Center, BrainLinks-BrainTools Cluster of Excellence, University of Freiburg, Freiburg D-79110, Germany.

^7^ Laboratory for Neuroengineering, Department of Health Sciences and Technology, Institute for Robotics and Intelligent Systems, ETH Zürich (ETH), Zürich, 8092, Switzerland.

^+^Equally Junior Contributors

^&^Equally Senior Contributors

^*^Corresponding Authors: Francesco m. Petrini (Francesco.petrini@sensars.com); Silvestro Micera (silvestro.micera@epfl.ch[santannapisa.it])

**Supplementary figures**


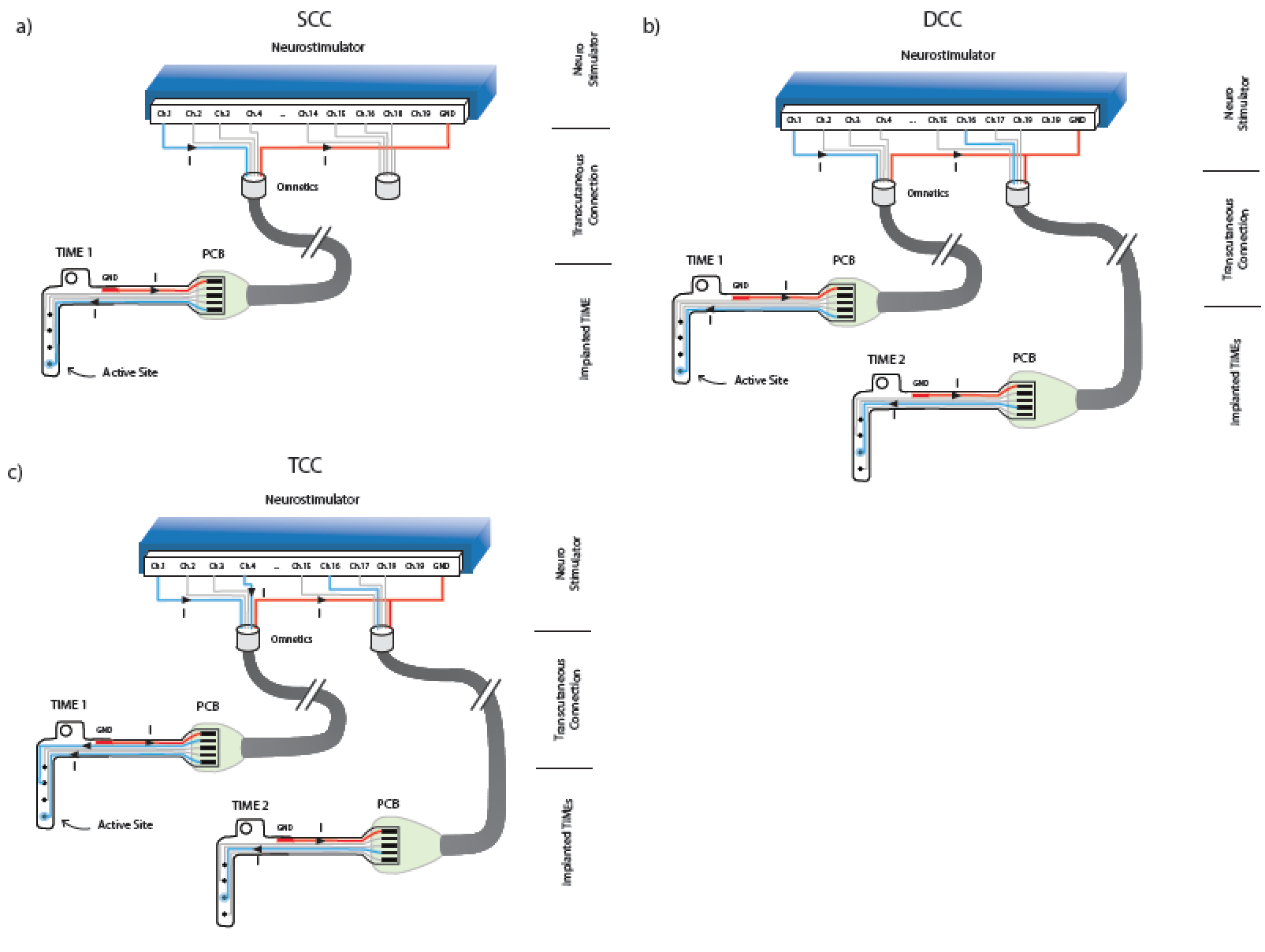


**Figure Supp. 1.** Scheme of single, double and triple channel configuration. SCC: the purple AS is active in respect to GND of TIME1. DCC: purple and green AS are active in respect to GND of TIME1. TCC: three ASs are active (purple, green and blue). Purple and green ones are stimulated in respect to GND of TIME1 while the blue AS stimulates in respect to GND of TIME2.


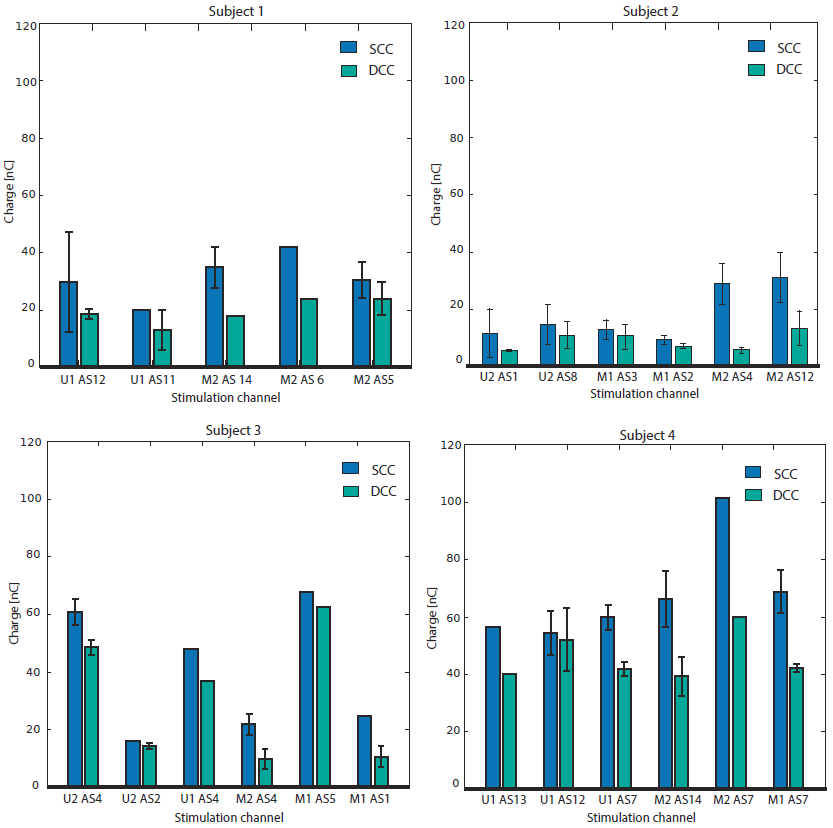


**Figure Supp. 2.** Perceptual threshold for all subjects according to the injected charge for SCC and DCC (N=30, 162, 30, 33, for Subject 1,2,3 and 4, respectively). Each stimulation channel used in SCC and in DCC is reported. The DCC combinations in which the channels reported in figure were used, are in Figure 2.

**Figure Supp. 3.** Subject 1 grand-average butterfly ERP and topographic scalp maps elicited by a sub-perceptual stimulation. N=197.

**
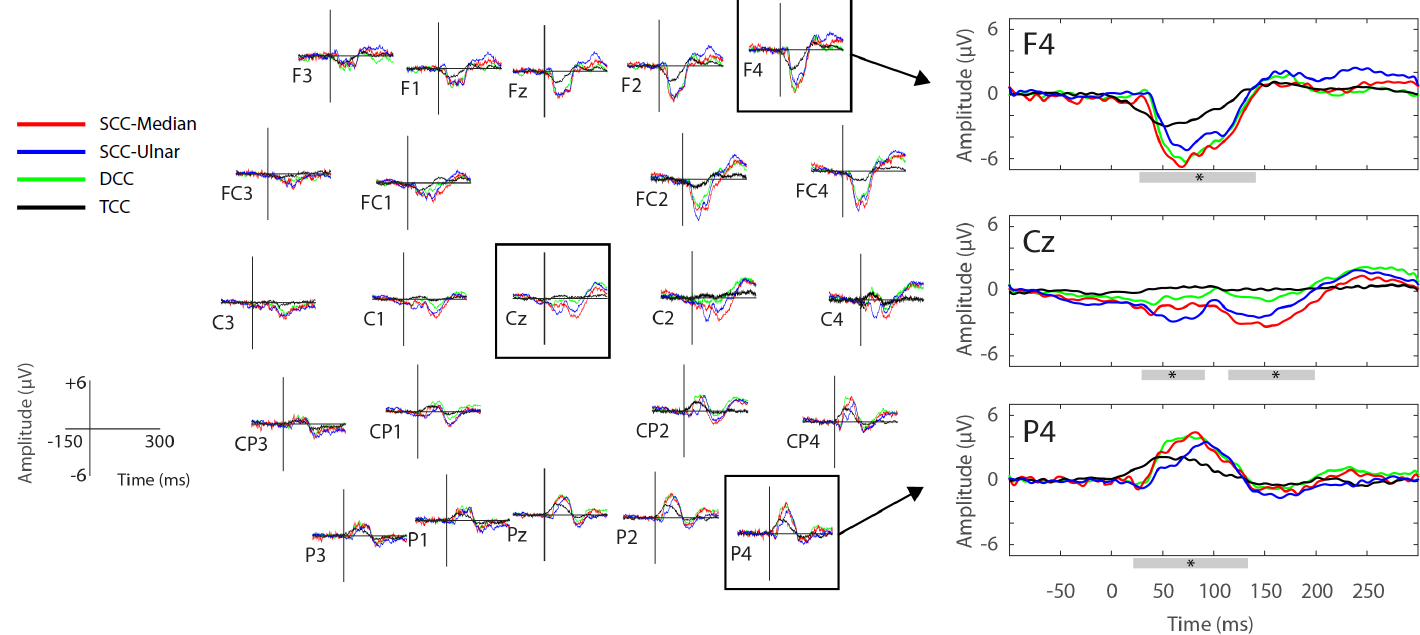
**

**Figure Supp. 4.** Subject 1 grand-average ERPs elicited by single channel configuration, bipolar and tripolar stimulation. Time intervals with significantly differences across conditions on three representative derivations (frontal – F4, central – Cz, posterior – P4) are marked with a * (p<0.05). N=412, 349, 427 and 337 respectively for SCC-median, SCC-ulnar, DCC, TCC.
